# Clinical Annotation Research Kit (CLARK): Computable Phenotyping Using Machine Learning

**DOI:** 10.2196/16042

**Published:** 2020-01-24

**Authors:** Emily R Pfaff, Miles Crosskey, Kenneth Morton, Ashok Krishnamurthy

**Affiliations:** 1 North Carolina Translational and Clinical Sciences Institute University of North Carolina at Chapel Hill Chapel Hill, NC United States; 2 CoVar Applied Technologies Durham, NC United States; 3 Renaissance Computing Institute University of North Carolina at Chapel Hill Chapel Hill, NC United States

**Keywords:** natural language processing, machine learning, electronic health records

## Abstract

Computable phenotypes are algorithms that translate clinical features into code that can be run against electronic health record (EHR) data to define patient cohorts. However, computable phenotypes that only make use of structured EHR data do not capture the full richness of a patient’s medical record. While natural language processing (NLP) methods have shown success in extracting clinical features from text, the use of such tools has generally been limited to research groups with substantial NLP expertise. Our goal was to develop an open-source phenotyping software, Clinical Annotation Research Kit (CLARK), that would enable clinical and translational researchers to use machine learning–based NLP for computable phenotyping without requiring deep informatics expertise. CLARK enables nonexpert users to mine text using machine learning classifiers by specifying features for the software to match in clinical notes. Once the features are defined, the user-friendly CLARK interface allows the user to choose from a variety of standard machine learning algorithms (linear support vector machine, Gaussian Naïve Bayes, decision tree, and random forest), cross-validation methods, and the number of folds (cross-validation splits) to be used in evaluation of the classifier. Example phenotypes where CLARK has been applied include pediatric diabetes (sensitivity=0.91; specificity=0.98), symptomatic uterine fibroids (positive predictive value=0.81; negative predictive value=0.54), nonalcoholic fatty liver disease (sensitivity=0.90; specificity=0.94), and primary ciliary dyskinesia (sensitivity=0.88; specificity=1.0). In each of these use cases, CLARK allowed investigators to incorporate variables into their phenotype algorithm that would not be available as structured data. Moreover, the fact that nonexpert users can get started with machine learning–based NLP with limited informatics involvement is a significant improvement over the status quo. We hope to disseminate CLARK to other organizations that may not have NLP or machine learning specialists available, enabling wider use of these methods.

## Introduction

Structured data in the electronic health record (EHR), such as diagnosis and procedure codes, numeric lab values, and admission and discharge dates, are extraordinarily valuable for development of computable phenotypes [1]. These are algorithms that translate clinical features into code that can be run against EHR data to define patient cohorts. Computable phenotypes can be used to efficiently identify potential study participants for recruitment, be shared among collaborators to enable multi-site cohort identification, or be posted publicly in repositories (eg, Phenotype KnowledgeBase) [2] for wide use. However, computable phenotypes that only make use of structured EHR data do not capture the full richness of a patient’s medical record, because they do not consider information found in the clinical notes.

National data networks such as the Electronic Medical Records and Genomics (eMERGE) network have demonstrated that unstructured, free-text clinical notes often contain critical information that is missing from the EHR's structured fields [3,4]. Social determinants of health, symptoms, and findings from imaging and pathology are among the features apt to be buried in free text. However, despite their importance, extraction of these features requires the use of more advanced informatics methods [5,6]. By making clinical note text more accessible, researchers can identify cohorts using inclusion or exclusion criteria typically captured only in notes and often available only through time-consuming, manual chart abstraction. While natural language processing (NLP) methods have shown success in extracting clinical features from text, current tools can be difficult to implement, require specialized technical knowledge to use, and entail extensive domain expertise for setup and validation [7,8]. Even with the existence of freely available NLP tools (eg, Apache’s cTAKES [9] and OpenNLP [10]), the use of such tools for computable phenotyping has been limited to research groups with substantial NLP expertise [11].

In the absence of this expertise, researchers are often obliged to perform time-intensive chart reviews on an overly inclusive set of patients to determine who qualifies for their study. This additional effort may increase costs and significantly lengthen the time between study start-up and participant recruitment. As an alternative to manual chart review, NLP augmented with machine learning can be used to identify cohorts where structured data is limited or not available, using the contents of free-text clinical notes [4-6,12-15]. We believe that the use of these technologies and methods need not be limited to informatics experts.

Computable phenotyping is a good fit for machine learning–based NLP, as phenotypes are essentially classification problems, as in, based on available information, a patient can be placed in an appropriate category (eg, positive or negative for a disease). A machine can be trained to extract and use features from unstructured data similarly to the way a physician can review a chart; both are methods to learn more about patients [4-6,12-15]. Machine learning–based NLP relies on clues found in clinical notes, which is closer to the process a clinician would employ in reviewing a chart than using structured data elements extracted from a clinical data warehouse.

Considering this need, our goal was to develop open-source phenotyping software that enables clinical and translational researchers to use machine learning–based NLP for computable phenotyping, without requiring deep informatics expertise. To meet this need, the North Carolina Translational and Clinical Sciences Institute, the University of North Carolina at Chapel Hill’s (UNC) National Institute of Health–funded Clinical and Translational Science Award, and CoVar Applied Technologies built CLARK (Clinical Annotation Research Kit) [16]. CLARK is specifically designed to be user-friendly, freely sharable, and applicable to a variety of translational research questions. CLARK is designed to take free-text clinical notes as input and classify those notes (and the associated patients) based on features (ie, words and phrases) defined by the user. At its core, CLARK is an approachable user interface to enable easier user interaction with scikit-learn [17], with features tailored towards interacting with clinical data and the needs of clinical researchers.

CLARK is designed to supplement, not replace, human effort [18] and judgment to reduce time spent conducting chart review, produce more robust computable phenotypes, and move studies to recruitment or data analysis more quickly. CLARK’s approach to adapting a highly technical methodology for use by nonexperts is a purposeful trade-off. It potentially sacrifices the exactitude of a years-long informatics study to increase the speed of development, ease of use, flexibility, and potential of reusability, while still accomplishing the end goal of a refined pool of potential study participants.

## Methods

CLARK enables nonexpert users to mine text using machine learning classifiers by specifying features for the software to match in clinical notes. It is best suited for performing cohort identification when criteria can be formulated as a classification problem (eg, differentiating between disease subtypes, symptomatic versus asymptomatic patients, and presence or absence of disease). Once the classification problem is identified, CLARK requires the user to start with a gold standard (or training corpus) of clinical notes provided by clinical subject matter experts. In the training corpus, the correct answer or classification is already known to the user and CLARK.

The process of creating a gold standard differs depending on the use case, but generally follows this pattern:

1. A patient cohort to be used as a gold standard is defined. This may be a cohort of patients already known to the investigator, patients in an existing registry for the condition of interest, or patients identified in a database query using as many structured data points as possible, and then manually chart-reviewed by the clinicians to identify which patients identified by the wide net are true cases.
2. If needed for the given use case, a matching set of patients without the condition of interest can be identified and used to serve as noncases in the gold standard.
3. The patients in the gold standard are divided into two sets for use as a training set and testing set. Some use cases divide 50/50, while others purposely oversample one or more classifications.
4. At our institution, policy dictates that a data analyst will then extract all clinical notes in a given period for the identified patients on behalf of the investigator. These notes are then converted to JSON format for loading into CLARK. One of the metadata fields for each note contains the true classification of the patient to whom it belongs, and this is what CLARK uses to train.

**Figure 1:**
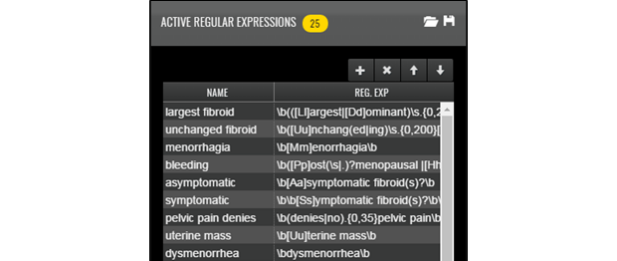
“Features” defined as regular expressions.

**Figure 2:**
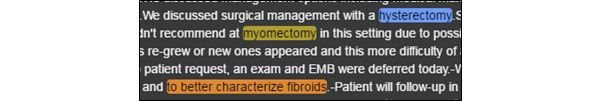
Highlighted feature matches.

Once loaded into CLARK, the user can browse through the notes in the corpus and define important features (words and phrases) in the gold standard using regular expressions or patterns to match. Expression matches are highlighted in a note browser for easy inspection. The user (a clinical subject matter expert) defines features that will give CLARK the information it needs to determine a given patient’s classification based on the contents of their notes, using logic similar to a physician performing a chart review. See Figure 1 for examples of features defined as regular expressions, in this case, to help CLARK identify patients with symptomatic uterine fibroids. See Figure 2 for examples of those features matched in a clinical note. Both positive and negative features can and should be defined, as the machine learning model will classify those patients matching “pelvic pain” and “denies pelvic pain” differently.

Once the features are defined, the CLARK user interface allows the user to choose from a variety of standard machine learning algorithms (linear support vector machine, Gaussian Naïve Bayes, decision tree, and random forest), cross-validation methods, and the number of folds (cross-validation splits) to be used in evaluation of the classifier.

Under the hood, CLARK contains a patient record processing engine that transforms the notes for each patient into a multi-dimensional feature vector based on the regular expression features defined by the user. For each sentence within a note, the number of matches for each regular expression is calculated. The vector of match counts is then summed across all sentences within a single note. Finally, the vectors are summarized at the patient level by calculating the mean feature vector across all of that patient’s notes. The user’s chosen machine learning algorithm is then able to consume these final patient-level feature vectors to train a model.

**Figure 3:**
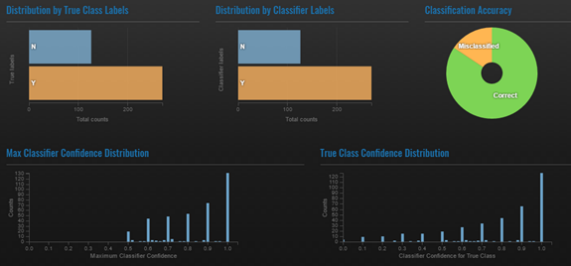
Interactive results dashboard.

After performing cross-validation on the training corpus, CLARK displays results in an interactive dashboard (Figure 3), which includes the classifier’s accuracy and confidence in each classification. The confidence scores are particularly helpful when iterating over a training set. If a user sees that CLARK is only 55% confident in many of its classifications, even if the classification is technically correct, that is an indicator that more or different features may be needed in the model to provide additional supporting data points. In a production-scale model, one could also use the confidence score to set a cut-off point to say that results would only be deemed reliable if they are at or above a certain confidence level.

Users can select individual patients (eg, the set of patients for whom CLARK was highly confident, but incorrect) to gather information to continue tuning the features used in training the model. The training process iterates as such until the user is satisfied with performance. At this point, a held-out testing set of labeled patients and notes can be processed using the pretrained algorithm. The user and CLARK are blinded to the correct labels of this held-out set. Once the model is run, the user can be unblinded to the labels in order to assess the model’s performance and calculate metrics such as sensitivity/specificity, F1-measure, and area under the receiver operating characteristic. The trained model can then be used to classify patients (and identify cohorts) in new, unannotated data.

CLARK has two primary components: A Python-based computation engine and a user interface built using Electron [19] and React [20]. All components of CLARK are themselves open-source, including the machine learning package, scikit-learn. CLARK runs well on personal computers and does not require a server or any other expensive information technology infrastructure to operate. The computation time required to train a model on a cohort of a few hundred patients generally takes just a few minutes, though this time is variable depending on the volume of notes. Moreover, CLARK does not require an internet connection to run, which means that (if desired) it can be set up on a computer or virtual machine quarantined from all network access. There is no physical or logical connection between CLARK and the institutional patient note repository (such as an enterprise data warehouse); instead, CLARK ingests an extract of patient notes that are provisioned to the research team. This extract can be stored locally on the same computer on which CLARK is installed (which would allow for the quarantine as mentioned earlier) or can be stored on a remote mount or network drive. This feature alleviates many institutions’ concerns regarding the security of open-source software on network-connected servers handling sensitive data and is a feature we included purposefully in anticipation of sharing the application.

Since its public release in 2017, CLARK has been used in several phenotyping applications at UNC, including efforts to classify patients with diabetes, uterine fibroids, nonalcoholic fatty liver disease (NAFLD), primary ciliary dyskinesia (PCD), cystic fibrosis, and bronchiectasis. The motivations for the use of CLARK for these particular phenotypes are presented in Textbox 1.

A selection of preliminary results from these studies are presented below.

**Use-case specific rationales for the use of CLARK.**

**Pediatric diabetes**

- International Classification of Diseases, Ninth Revision (ICD-9) diagnosis codes (the standard at the time this study was ongoing) for pediatric diabetes are fairly sensitive, but less specific when determining the presence or absence of diabetes in a patient, as patients may be given codes for diabetes if they have, for example, diabetes risk factors [21]. Incorporating clinical notes in the phenotype provides another, more specific source of information to help identify true cases.

**Symptomatic uterine fibroids**

- Women in whom uterine fibroids are identified will have an ICD-9 or International Classification of Diseases, Tenth Revision (ICD-10) diagnosis code for the condition recorded in their EHR, regardless of whether the fibroids are symptomatic. Thus, using only structured data in a fibroid computable phenotype identifies many asymptomatic women who would not qualify for this particular study [22]. Using clinical text as part of the phenotype is a way to account for symptoms in addition to the presence of fibroids.

**Nonalcoholic fatty liver disease**

- NAFLD does not have reliable ICD-9 or ICD-10 diagnosis codes and is underdiagnosed [23]. However, characteristics of NAFLD can be gleaned from clinical notes to identify patients missed with structured data algorithms.

**Primary ciliary dyskinesia**

- There is no specific ICD-9 or ICD-10 diagnosis code for PCD, meaning that this cohort cannot be identified using structured data alone. A combination of factors appearing in clinical notes can, when taken together, identify these patients with more certainty.

## Results

The results shown in Tables 1 and 2 are preliminary or early results for computable phenotyping studies in which CLARK has been applied. We present these results here to provide a picture of CLARK’s potential utility for these and other phenotyping exercises. Each of these examples used CLARK’s random forest option and were tested using 10-fold cross-validation. Note that study 1 (pediatric diabetes) used an early version of CLARK, well before its 2017 public release. The remaining studies all used the newest public version of CLARK.

**Table 1:** Select studies using CLARK for computable phenotyping.

Research question	Example features	Example regular expressions
Among a set of pediatric patients identified as potentially diabetic using structured data, can we use the patients’ clinical notes to identify the true positive cases [24]?	“Type 1 diabetes” “Insulin-dependent diabetes”	\bDM\W*T?(1|I)\b|\bT(ype)?\W*(1|I)\W*DM|\IDDM \b*insulin\W+depend\w+
Among a set of women with an ICD-9^a^ diagnosis code for uterine fibroids, can we use free-text reports from MRIs^b^ and ultrasounds to determine which patients are symptomatic, versus asymptomatic [25]?	“Significant fibroids” “Denies pelvic pain” “Vaginal bleeding”	([Mm]ultiple |[Pp]rominent |[Ll]arge )([Uu]terine |[Ii]ntramural )?fibroid(s)? (denies|no).{0,35}pelvic pain ([Pp]ost(\s|.)?menopausal |[Hh]eavy |[Aa]bnormal |[Ee]xtended )(vaginal )?bleeding\s.{1,750}fibroid(s)?
Among a set of patients with biopsy-proven NAFLD^c^, non-NAFLD liver disease, and healthy controls, can we use the patients’ clinical notes to differentiate the NAFLD patients from the other, similar conditions and healthy controls [23]?	BMI^d^≥40 (body mass index) “NAFLD”	((bmi|body\smass\sindex|bmi)?\scalculated)[\s\w:]{0,7}(([4][0-9].?[0-9]?[0-9]?)|([5][0-9].?[0-9]?[0-9]?)|([6][0-9].?[0-9]?[0-9?)|([7][0-9].?[0-9]?[0-9]?)|([8][0-9].?[0-9]?[0-9]?)) ((NAFLD|((non[0]?alcoholic)?\sfatty\sliver\s(disease)?)|K76\.0))
Among a set of patients with known PCD^e^, cystic fibrosis, bronchiectasis, and healthy controls, can we use the patients’ clinical notes to differentiate the PCD patients from the other, similar conditions and healthy controls? (Work ongoing.)	“Situs inversus” “Denies shortness of breath” “Ear tubes”	(s|S)itus (inversus|ambiguous)|(d|D)extrocardia|(h|H)eterotaxy (without|(N|n)o\b|(N|n)egative|(D|d)enies).{1,25}shortness of breath (E|e)ar tubes?|tympanoplasty|P\.?E\.? tubes?

^a^ICD-9: International Classification of Diseases, Ninth Revision.

^b^MRI: magnetic resonance imaging.

^c^NAFLD: nonalcoholic fatty liver disease.

^d^BMI: body mass index.

^e^PCD: primary ciliary dyskinesia.

**Table 2:** Evaluating performance of the research questions of each study.

Base population (n)	Classifications (true n from gold standard)	Model Performance
Pediatric patients identified by a wide-net structured EHR^a^ data algorithm [21] as having possible diabetes (1348)	True positive case (537) versus false positive case (811)	Sensitivity=0.91; Specificity=0.98
Women with uterine fibroids identified by a structured EHR data algorithm [22] (163)	Symptomatic fibroids (120) versus asymptomatic fibroids (43)	Positive predictive value=0.81; Negative predictive value=0.54
Patients with biopsy-proven NAFLD^b^, non-NAFLD liver disease, and healthy controls (55)	NAFLD cases (19) versus a mix of non-NAFLD liver disease cases and healthy controls (36)	Sensitivity=0.90; Specificity=0.94
Research registry of patients with confirmed PCD^c^, cystic fibrosis, or bronchiectasis, as well as healthy controls (247)	PCD case (22) versus a mix of CF^d^ cases, bronchiectasis cases, and controls (225)	Sensitivity=0.88; Specificity=1.00

^a^EHR: electronic health record.

^b^NAFLD: nonalcoholic fatty liver disease.

^c^PCD: primary ciliary dyskinesia.

^d^CF: cystic fibrosis.

## Discussion

### Primary Results

Our findings demonstrate CLARK’s potential to enhance the ability to define computable phenotypes for cohorts that require going beyond structured EHR data. Using clinical “clues” provided by clinical subject matter experts, CLARK was able to identify concepts in free-text notes that are either unreliable or not present in structured data.

This is the first time these CLARK-specific results have been published outside of abstracts; thus, these algorithms have not yet been unleashed on data beyond the training and test sets. Running the PCD or NAFLD algorithms on UNC’s entire clinical data warehouse, for example, would be a true test of these phenotypes’ utility. Once we take this step, there is a strong chance that we could identify previously undiagnosed (or uncoded) cases of these diseases, which could have a direct impact on patients’ lives.

One consistent feature of machine learning and natural language processing is that 100% accuracy is exceedingly rare, except by chance (or by overfitting one’s model). As a result, clinicians must tolerate many false positives and false negatives, with the level of tolerance based on the use case. Because CLARK outputs a confidence level with each of its classification decisions, users have the flexibility to, for example, only accept CLARK’s classifications when the confidence is above a certain cut-off point and opt to review the rest manually. This option may engender more trust in CLARK’s results, while still cutting down on the number of charts needed to be reviewed manually.

### User-Friendliness as Innovation

Our intention for CLARK’s interface to be accessible to less technical users is itself an innovation. While NLP is a well-established informatics method in health care and translational research, its use is generally limited to experts with the requisite technical knowledge and programming skills [11]. While the same could be said for many methodologies (eg, some advanced statistical analysis may be limited to biostatisticians), one key aspect of NLP makes democratization particularly desirable: the requirement that machine learning–based NLP models be trained before applying them to new data. In the health care context, this means training a model to mimic clinical inference. For that reason, clinicians, not informaticians, are best suited to train models. However, at present, only informaticians are capable of executing and iterating through the training process. CLARK is designed to address that gap. Though we have not done a formal usability study at this time, design decisions during application development were made with our intended audience (noninformatician clinician-scientists) in mind.

CLARK’s most technical prerequisite is a basic understanding of regular expressions or snippets of text that define a pattern of alphanumeric characters. While the most complex regular expressions are not likely to be used by nonexperts, we have had success training clinician-researchers to build simple regular expressions and use them in CLARK on their own. In early user testing, we successfully taught basic regular expression syntax in a one-hour session to approximately ten investigators who were initially unfamiliar with the concept. Yes, regular expressions can be tricky for even experienced programmers, and these one-hour training sessions are not intended to result in mastery. Instead, these sessions enable investigators to start basic pattern matching (eg, “(D|d)iabetes”). When more complex expressions are needed, our informatics team is available to assist, while still allowing the investigator to use the software and do their analysis independently.

Once that knowledge is gained, learning how to build a basic model in CLARK takes only minutes. In three of the four studies described in our Results, the clinician investigators worked side-by-side with an informatician in the CLARK user interface to browse through notes and define regular expressions as a team. Additionally, we have examples of ongoing studies in which the clinician investigators are using CLARK mostly on their own (eg, to identify breast cancer subtypes), with only a small amount of support from an informatician. The most common questions we receive from investigators are not around regular expressions, but rather what is happening within the black box of the machine learning model. We have found that the idea of a machine making decisions that are opaque to the human user is a challenging concept to explain in lay language and is something we continue to work on. Regardless, the fact that nonexpert users can get started with machine learning–based NLP with limited informatics involvement is a significant improvement over the status quo.

### Conclusions

We believe that CLARK has enormous potential to allow more complex cohorts to be identified using computable phenotyping, by unlocking the valuable content of free-text clinical notes and other unstructured data. Moreover, by making the user interface understandable to noninformaticians, yet maintaining a sophisticated backend capable of running complex models, CLARK achieves what most existing machine learning–based NLP applications do not [7]: user-friendly design that supports the interdisciplinary nature of NLP. By making CLARK open source, we hope to disseminate CLARK to other sites that may not have NLP or machine learning specialists available, enabling wider use of these methods, and spurring innovation and collaboration in computable phenotyping.

## Abbreviations

CLARK: Clinical Annotation Research Kit
EHR: electronic health record
eMERGE: Electronic Medical Records and Genomics network
ICD-9: International Classification of Diseases, Ninth Revision
ICD-10: International Classification of Diseases, Tenth Revision
NLP: natural language processing
PCD: primary ciliary dyskinesia
NAFLD: nonalcoholic fatty liver disease
